# Effects of nuclear factor-κB signaling pathway on periodontal ligament stem cells under lipopolysaccharide-induced inflammation

**DOI:** 10.1080/21655979.2022.2051690

**Published:** 2022-03-17

**Authors:** Mingyue Chen, Xiaobo Lin, Li Zhang, Xiaoli Hu

**Affiliations:** a Department of Stomatology, Taihe Hospital, Hubei University of Medicine, Shiyan, Hubei Province, China; b Department of Rehabilitation, Taihe Hospital, Hubei University of Medicine, Shiyan, Hubei Province, China

**Keywords:** Periodontitis, NF-κB signaling pathway, lipopolysaccharide (LPS), periodontal ligament stem cells, osteogenic differentiation

## Abstract

Lipopolysaccharide (LPS) induces inflammatory stress and apoptosis. This study focused on the effect of nuclear factor kappa B (NF-κB) signaling pathway on proliferation and osteogenic differentiation of human periodontal ligament stem cells (hPDLSCs) after LPS induction and its mechanism. We first isolated hPDLSCs from human tooth root samples *in vitro*. Then, flow cytometry detected positive expression of cell surface antigens CD146 and STRO-1 and negative expression of CD45, suggesting the hPDLSCs were successfully isolated. LPS significantly induced increased apoptosis and diminished proliferation of hPDLSCs. The NF-κB pathway agonist phorbol 12-myristate 13-acetate (PMA) or p65 overexpression inhibited the proliferation of LPS-treated hPDLSCs and promoted apoptosis. PMA also promoted LPS-induced up-regulation of the expression of inflammatory factors TNF-α and IL-6 and down-regulation of the expression of anti-inflammatory factor IL-10. Additionally, LPS was confirmed to lead to a reduction of alkaline phosphatase (ALP) activity, calcium nodules, and expression of osteogenic markers Runt-related transcription factor 2 (Runx2) and osteopontin. This reduction could be promoted by PMA. Western blotting further indicated that PMA could promote LPS-induced decrease of expression of p65 (cytoplasm), and total cellular proteins IKKα and IKKβ in hPDLSCs, while protein expression of p-IκBα (cytoplasm) and p65 (nucleus), and p-IκBα/IκBα ratio was elevated. By contrast, inhibition of the NF-κB pathway (PDTC) or small-interfering RNA targeting NF-κB/p65 (p65 siRNA) showed the opposite results. In conclusion, activation of NF-κB signaling in LPS-induced inflammatory environment can inhibit the proliferation and osteogenic differentiation of hPDLSCs. This study provides a theory foundation for the clinical treatment of periodontitis.

## GRAPHICAL ABSTRACT

**Figure:**
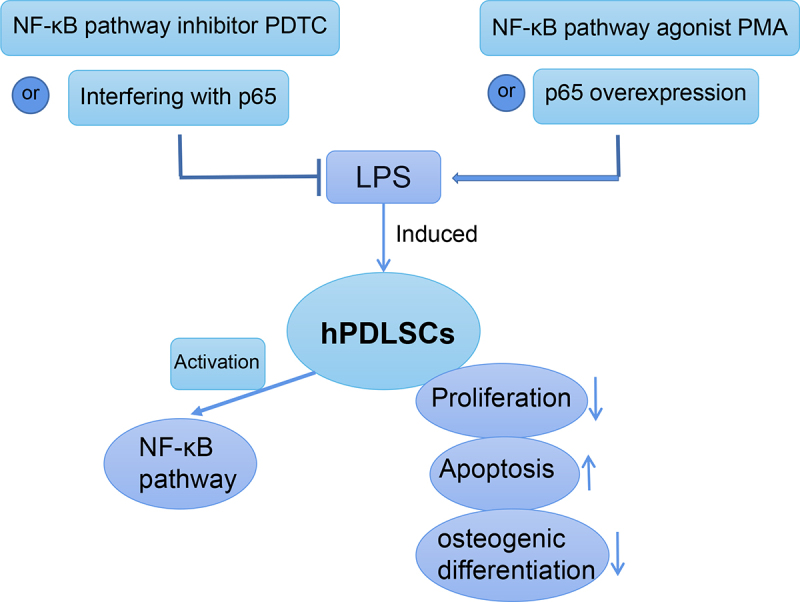

## Introduction

Periodontitis, in modern society, is increasingly common in the adult population due to life and work stresses. According to the reports, the prevalence of periodontitis is more than 65% in adults over 40 years old, while it is as high as 85% in those over 60 years old [1]. Among the many factors leading to this oral inflammatory disease, the main cause is the destruction of immune response of the body, which resulting in the excessive reproduction of specific bacteria or pathogens and consequently destruction of periodontium [2]. Bacteria in the oral cavity and the host are in a dynamic balance in normal subjects. But once the balanced is destroyed, periodontal pathogens become the predominant bacteria, triggering the immune response and inflammatory response of the host and the production of a large number of inflammatory mediators [3]. These inflammatory mediators not only further destroy periodontium but also induce a systemic inflammatory response in the body through blood circulation [4]. It has been pointed out that excessive inflammatory factors are inducers of many systemic diseases and seriously threaten systemic health [5].

Lipopolysaccharide (LPS) is a crucial virulence factor participating in the pathogenesis of periodontitis [6]. LPS can be recognized by pattern recognition receptor such as TLR4 in the body, which can activate the body’s immune response to induce the infiltration of a lot of inflammatory cells in periodontium. Meanwhile, LPS promotes the release of pro-inflammatory cytokines such as IL-1 and IL-6 while inhibiting the production of anti-inflammatory cytokines such as IL-10 [7]. Thus, LPS induction can destroy local periodontium and accelerate the progression of periodontitis.

It has been demonstrated that the inflammatory microenvironment in patients with periodontitis inhibits the proliferation and osteogenesis of human periodontal ligament stem cells (hPDLSCs), thereby affecting the regeneration and remodeling of periodontal tissue, gingiva, and alveolar bone [8]. hPDLSCs are a class of mesenchymal stem cells derived from the periodontal membrane with high proliferative, self-regenerative and regenerative capacities [9]. As early as 2004, studies have reported that there are stress sensors on the surface of PDLSCs, which can differentiate into specific adult cells when stimulated and are important seed cells for periodontium reconstruction and regeneration [10]. For example, hPDLSCs can be induced to differentiate into adipocytes, cementoblast-like cells, and osteoblasts in vitro [11]. Additionally, they have more growth potential than bone marrow mesenchymal stem cells [12]. Therefore, hPDLSCs often serve as seed cells for tissue engineering [13]. In treatment of periodontitis, the implanted cells will face a complex inflammatory microenvironment, which hinders the biological functions of hPDLSCs. Studies have shown that PDLSCs derived from the inflammatory environment have reduced osteogenic differentiation and adipogenic differentiation ability and impaired immunoregulation [14]. Another study has speculated that the inhibition of hPDLSCs biological characteristics by inflammatory environment is related to nuclear factor kappa B (NF-κB) [15], but this speculation is still undecided. In this study, we induced an inflammatory microenvironment by LPS to explore the effects of NF-κB signaling pathway on hPDLSCs proliferation and osteogenic differentiation. This study aims to provide effective information of molecular relationship and theoretical basis for the clinical treatment of periodontitis or for the clinical translation of periodontal tissue engineering.

## Materials and methods

### Tooth sample collection

Tooth samples were collected from healthy patients who underwent orthodontic extraction in Maxillofacial Surgery in our hospital. All patients have no tooth decay, apical periodontitis, periodontitis, and no systemic diseases. hPDLSCs were isolated from the obtained tooth samples. In addition to the patients’ informed consent forms, an approval for this study was obtained from the Ethics Committee of Taihe Hospital, Hubei University of Medicine (Approval number: 2020KS002).

### Isolation of hPDLSCs

The collected tooth root samples were rinsed three times with sterile PBS buffer. Then, periodontal ligament tissues at the one-third part of the root were scraped using a scalpel blade, and the obtained periodontal tissues were cut into 1 mm^3^ tissue blocks with ophthalmic scissors. Subsequently, 1-h digestion of the tissues was performed with 3 mg/mL type I collagenase at 37°C and terminated using DMEM complete medium. Next, after centrifugation for 5 min at 4°C and 800 r/min, the supernatant was aspirated. On completion of addition of complete medium, the tissue blocks were transferred to the bottom of a culture flask with an area of 25 cm^2^, and then placed in an incubator for culture. The culture flask was inverted 4 h later and the migration of cells around the tissue block was observed every 3 days. When the cell density reached 80%–90%, 0.25% trypsin was used for digestion and passage to obtain the first passage of hPDLSCs.

### Culture and treatment of hPDLSCs

The above-obtained first passage of hPDLSCs in logarithmic growth phase was digested using trypsin. By using a limiting dilution analysis, the cells in the cell suspension were counted and then were plated in 96-well plate (5 × 10^3^ cells/well) for the following 5-day culture at 37°C in 5% CO_2_ incubator (Thermo, USA). After that, the wells containing single-cell clones were added 0.1 mL of complete culture medium for continuous culture. When one-half of the bottom of the wells was covered with the cells, routine passage was carried out and third passage cells were taken for subsequent experiments.

According to different treatments, hPDLSCs were split into Control group, LPS group (1 μg/mL LPS) [16], LPS+PMA group (1 μg/mL LPS + 100 nM NF-κB agonist phorbol-12-myristate-13-acetate) [17], and LPS+PDTC group (1 μg/mL LPS + 10 μM NF-κB inhibitor pyrrolidine dithiocarbamate) [18]. LPS (from *Escherichia coli*) [19], PMA, and PDTC were obtained from Sigma-Aldrich (St. Louis, MO, USA). In the latter two groups, hPDLSCs were pretreated with PMA or PDTC for 30 min, followed by stimulation with LPS.

### Osteoblast differentiation of hPDLSCs

The hPDLSCs in each group were treated for 24 h and then plated in 6-well plates (2 × 10^4^ cells/mL), respectively. With 80% confluency, the original culture medium was aspirated, followed by with PBS wash. Subsequently, osteogenic induction medium supplemented with 10 mmol/L β-glycerophosphate, 50 µg/mL ascorbic acid, and 10 nM dexamethasone was added, and the medium was replaced every 3 days. After 2 weeks, the cells were fixed with 4% paraformaldehyde and then stained with alkaline phosphatase or alizarin-red to determine whether osteogenic differentiation was successful.

### Flow cytometry

First passage of isolated hPDLSCs were digested with 0.25% trypsin and then counted to adjust the density to 1 × 10^6^ cells/mL. The antibodies to hPDLSCs surface antigens were diluted with precooled PBS buffer according to the instructions, including CD146 (eBioscience, USA), STRO-1 (eBioscience, USA), and CD45 (eBioscience, USA). The antibodies were added to the cells for 1 h incubation in the dark. Finally, precooled PBS buffer was used to rinse the cells for three times and then a flow cytometer for detection of hPDLSCs surface antigen markers.

For cell apoptosis detection, cells after 48 h of treatment in each group were digested using trypsin, followed by centrifugation (800 r/min, 5 min, 4°C) and concentration adjustment to 5 × 10^5^ cells/mL by PBS. Then, 200 μL of cell suspension were taken to add 10 μL of AnnexinV-FITC, and 10 μL of 20 mg/L PI solution, and subsequently 10 min incubation was performed in the dark at ambient temperature. After that, 500 μL of PBS was added, and finally apoptosis was detected by the flow cytometer.

### Cell counting kit-8 assay

Each group of hPDLSCs were seeded in 96-well plates at a density of 5 × 10^3^ cells/well. After an incubation for 24, 48, and 72 h, respectively, 10 uL of cell counting kit-8 (CCK8) reagent (Beyotime Biotechnology, China) was added into each well, and incubated for 4 h. With a microplate reader for measurement of the absorbance (450 nm), cell proliferation rate was finally calculated.

### Enzyme-linked immunosorbent assay

The cell culture medium of each group was collected for centrifugation (1000 r/min, 10 min, 4°C) so as to remove cells or cell debris. After that, new sterile centrifuge tubes were utilized to collect the supernatant. Evaluation of TNF-α, IL-6, and IL-10 expression in the medium was completed on the basis of the enzyme-linked immunosorbent assay (ELISA) kit instructions (Nanjing Jiancheng Bioengineering Institute, China).

### Alkaline phosphatase staining

The culture medium of hPDLSCs in each group was removed at 7 days of inducing osteogenic differentiation. Then, PBS solution was adopted for rinsing the cells three times at 37°C, followed by 30 min fixation step using 4% paraformaldehyde. Then, the fixative was removed and the cells were rinsed three times with PBS again. On completion of preparation of the staining solution using BCIP/NBT alkaline phosphatase (ALP) staining reagent (Beyotime Biotechnology, China), the cells were stained and incubated in the dark for 30 min. Following the removal of the staining solution, PBS was utilized to rinse the cells three times. Eventually, the images were obtained with a Canon camera.

### Alizarin-red staining

hPDLSCs were cultured in 6-well plates to achieve 80% confluency. Then, osteogenic differentiation induction medium was added and culture was continued for 21 days. After cell culture, PBS solution was adopted for rinsing the cells three times, followed by 15 min fixation step using 4% paraformaldehyde. Subsequently, each well was added with an appropriate amount of 2% alizarin-red staining solution (Nanjing Jiancheng Bioengineering Institute, China). Following the removal of the staining solution, PBS was utilized to rinse three times. Finally, the images were obtained with a Canon camera. For quantification, the alizarin red staining solution was extracted with 10% cetylpyridinium chloride (Sigma-Aldrich, USA) for 10 min and the absorbance value at 550 nm was determined using an enzyme marker [20].

### Western blotting

Cells were seeded in 6-well plates. After various treatment, the nucleus and cytoplasm were extracted using nuclear protein extraction kit, and the total protein was extracted with RIPA buffer. The concentration of proteins was determined using BCA assay kit (Abcam, UK). Equal proteins were separated by 12% SDS-PAGE, and then transferred to PVDF membranes. After blocked with 5% nonfat milk for 1 h at room temperature, the membranes were incubated with primary antibodies Runx2 (Abcam, UK), Osteopontin (Abcam, UK) at 4°C overnight. Next, secondary antibodies were added for another 1 h incubation and then the membranes were rinsed for another three times. After that, protein bands were identified with enhanced chemiluminescence reagent (ECL, Thermo, USA) and were visualized using the ChemiScope Western Blot Imaging System. The gray value analysis was performed by using ImageJ software. The relative expression of extracted proteins was calculated, with β-actin as the internal reference for extracted whole cell or cytoplasmic proteins and H3 as the internal reference for extracted nuclear proteins.

### Statistical analysis

By using SPSS 25.0, one-way analysis of variance and independent sample t-test were performed. Experimental results were all expressed in the form of mean ± standard deviation (SD). *P* < 0.05 was the criterion for significance of differences.

## Results

### Identification of hPDLSCs

**Figure 1.**
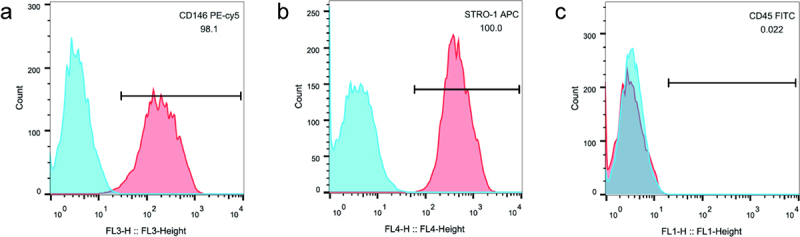
Detection of surface markers of hPDLSCs. CD146 antigen expression (a), STRO-1 antigen expression (b), CD45 antigen expression (c) on the hPDLSCs surface. hPDLSCs, human periodontal ligament stem cells.

Flow cytometry showed the positive results of hPDLSCs surface antigens CD146 (Figure 1(a)) and STRO-1 (Figure 1(b)), and the negative result of CD45 (Figure 1(c)). Taken together, it was proved that the cells isolated in this study were hPDLSCs.

### Effect of NF-κB pathway on LPS-induced proliferation and apoptosis of hPDLSCs

**Figure 2.**
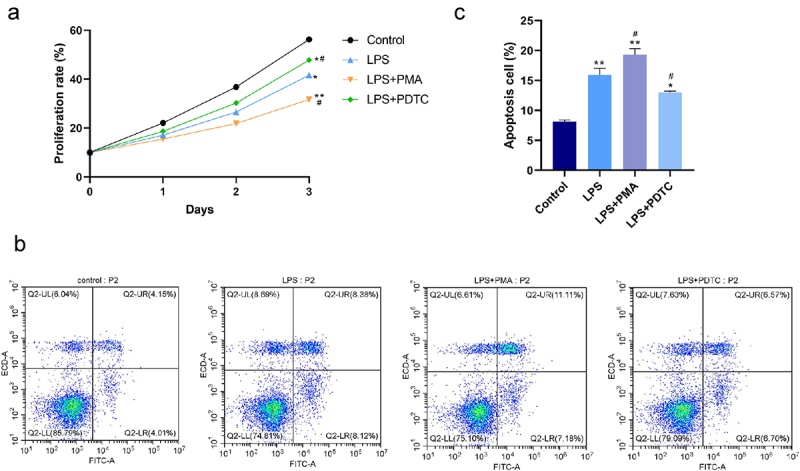
Effect of NF-κB pathway on proliferation and apoptosis of LPS-induced hPDLSCs. A: Detection of proliferation of hPDLSCs by CCK-8 assay; B-C: Detection of apoptosis of hPDLSCs by flow cytometry. hPDLSCs, human periodontal ligament stem cells; LPS, Lipopolysaccharide; PMA, NF-κB pathway agonist; PDTC, NF-κB pathway inhibitor. *P < 0.05 and **P < 0.01 *vs*. control group, ^#^*P* < 0.05 *vs*. LPS group.

As shown in the CCK-8 and flow cytometry results (Figure 2(a-c)), LPS resulted in significant inhibition of proliferation and promotion of apoptosis of hPDLSCs. NF-κB agonist PMA promoted the above-described effects of LPS. That is, a further decrease of proliferation rate and an increase of apoptosis rate were found in the LPS+PMA group, while opposite changes were found in the LPS+PDTC group. Collectively, promoted proliferation and suppressed apoptosis in hPDLSCs treated with LPS were shown as a result of the inhibition of NF-κB signaling pathway activation.

### Effect of NF-κB pathway on cytokines in LPS-induced hPDLSCs

**Figure 3.**
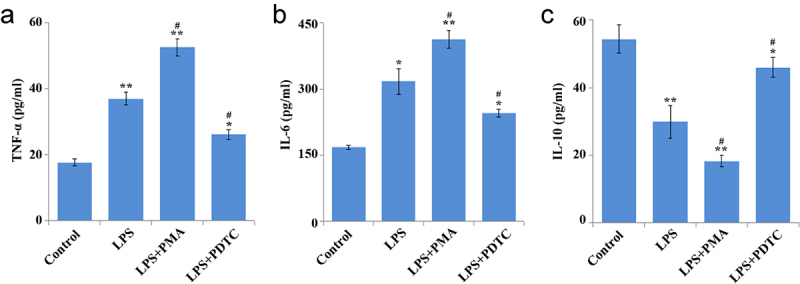
Effect of NF-κB pathway on cytokines in hPDLSCs after LPS induction. Expression of TNF-α (a), IL-6 (b), IL-10 (c) in LPS-treated hPDLSCs. hPDLSCs, human periodontal ligament stem cells; LPS, Lipopolysaccharide; PMA, NF-κB pathway agonist; PDTC, NF-κB pathway inhibitor; *P < 0.05 and **P < 0.01 *vs*. control group, ^#^*P* < 0.05 *vs*. LPS group.

As shown in the ELISA results (Figure 3(a-c)), LPS caused a marked increase of TNF-α and IL-6 expression and decrease of IL-10 expression. In comparison with the LPS group, significant up-regulation of TNF-α and IL-6 expression and down-regulation of IL-10 were found in the LPS+PMA group, while opposite changes were found in the LPS+PDTC group. Collectively, lower TNF-α and IL-6 expression and higher IL-10 expression in hPDLSCs treated with LPS were shown as a result of the inhibition of NF-κB signaling pathway activation.

### Effect of NF-κB signaling pathway on osteogenic differentiation of LPS-induced hPDLSCs

**Figure 4.**
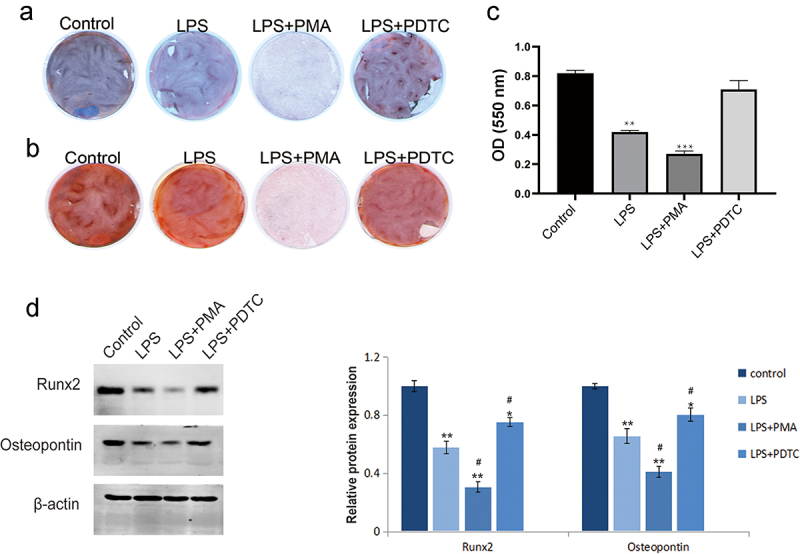
Effect of NF-κB signaling pathway on osteogenic differentiation of hPDLSCs after LPS induction. a: ALP staining of hPDLSCs; b-c: Alizarin-red staining of hPDLSCs and its quantitative analysis; d: Western blotting-based detection of the expression of Runx2 and osteopontin in hPDLSCs. hPDLSCs, human periodontal ligament stem cells; LPS, Lipopolysaccharide; PMA, NF-κB pathway agonist; PDTC, NF-κB pathway inhibitor. *P < 0.05, **P < 0.01 and ***P < 0.001 *vs*. control group; ^#^*P* < 0.05 *vs*. LPS group.

As shown in ALP staining (Figure 4(a)) and alizarin-red staining (Figure 4(b,c)), LPS caused significant decreases of ALP activity, degree of ossification, and calcium nodules. Co-treatment of LPS and PMA further promoted the above-mentioned effects of LPS, while the opposite changes were found in the LPS+PDTC group. In addition, western blotting (Figure 4(d)) revealed a reduction of the expression of osteogenic differentiation-related proteins Runx2 and osteopontin after LPS induction; PMA promoted this reduction while PDTC had the opposite effect. These results confirmed that the ability of osteogenic differentiation of hPDLSCs was inhibited by LPS, while it could be increased after inhibition of NF-κB pathway.

### Expression of NF-κB pathway-related proteins in LPS-induced hPDLSCs

**Figure 5.**
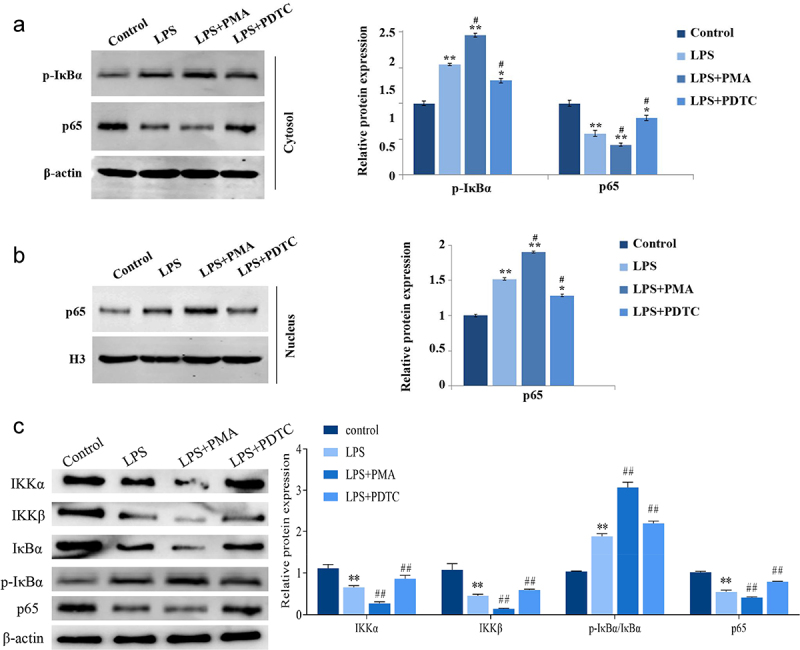
Expression of NF-κB signaling pathway-related proteins in hPDLSCs after LPS induction. Proteins expression of p-IκBα and p65 in the cytoplasm (a), p65 in the nucleus (b), and IKKα, IKKβ, p-IκBα, IκBα and p65 in human periodontal ligament stem cells (c) were detected with Western blot. hPDLSCs, human periodontal ligament stem cells; LPS, Lipopolysaccharide; PMA, NF-κB pathway agonist. PDTC, NF-κB pathway inhibitor. *P < 0.05 and **P < 0.01 *vs*. control group, ^#^*P* < 0.05 *vs*. LPS group.

LPS significantly induced the expression of p-IκBα (cytoplasm) and p65 (nucleus) and inhibited the expression of p65 (cytoplasm) and total cellular proteins IKKα and IKKβ in hPDLSCs, and the ratio of p-IκBα/IκBα was significantly higher (Figure 5(a-c)). PMA activated NF-κB signaling pathway and enhanced the LPS-caused promotion or inhibition of NF-κB pathway-related proteins in hPDLSCs. And PDTC, an inhibitor of NF-κB signaling pathway, reversed the effects of LPS on expression of the above proteins (Figure 5(a-c)).

### Effect of interfering with p65 expression on the proliferation and osteogenic differentiation of LPS-induced hPDLSCs

**Figure 6.**
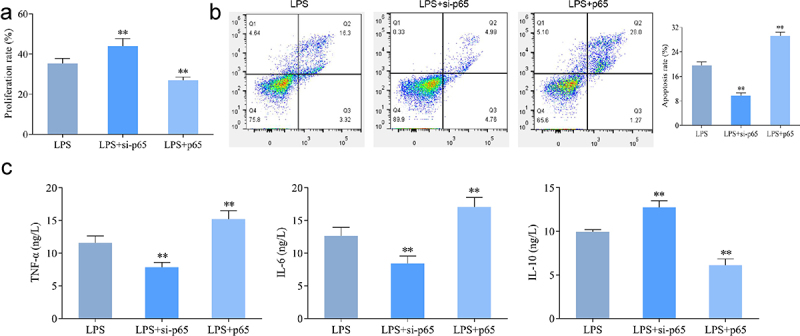
Effect of intervention of p65 expression on the proliferation of hPDLSCs and inflammatory factors after LPS induction. a: CCK8 assay for proliferation of hPDLSCs; b: Flow assay for apoptosis of hPDLSCs. c: ELISA for TNF-α, IL-6 and IL-10 levels in the supernatant of hPDLSCs. hPDLSCs, human periodontal ligament stem cells. **P < 0.01 *vs*. LPS group.

Moreover, we directly and specifically regulate NF-κB expression by interfering with or overexpressing p65, thus further validating the role of NF-κB signaling pathway in LPS-induced hPDLSCs. As shown in Figure 6(a,b), interfering with p65 expression significantly upregulated proliferation of LPS-treated hPDLSCs and inhibited their apoptosis. In contrast, after overexpression of p65, hPDLSCs proliferation was inhibited and apoptosis was increased. ELISA results showed that interference with p65 expression caused significantly decreased levels of TNF-α and IL-6, and increased levels of IL-10 in the supernatants of LPS-induced hPDLSCs, while overexpression of p65 showed the opposite trend (Figure 6(c)).

**Figure 7.**
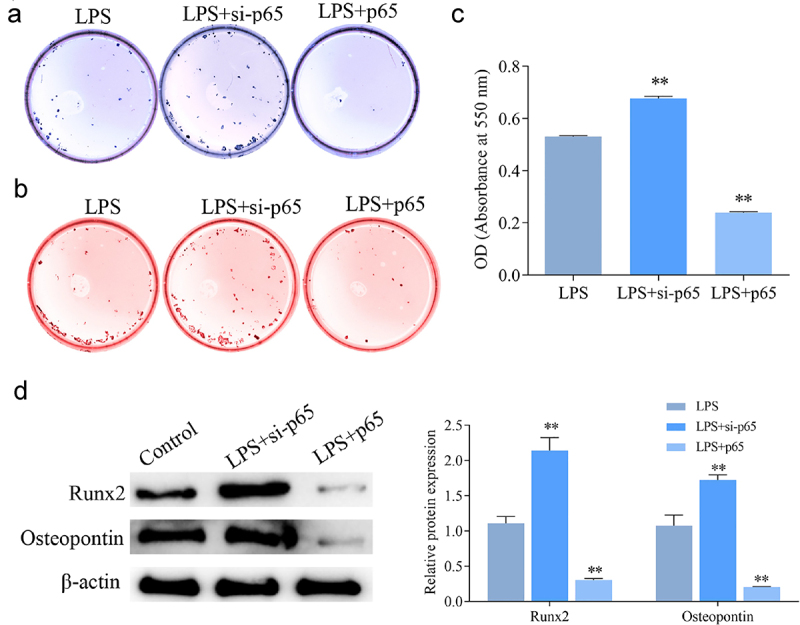
Effect of intervention of p65 expression on osteogenic differentiation of hPDLSCs after LPS induction. a: ALP staining of hPDLSCs; b-c: Alizarin-red staining of hPDLSCs and its quantitative analysis; d: Western blotting-based detection of the expression of Runx2 and osteopontin in hPDLSCs. hPDLSCs, human periodontal ligament stem cells. **P < 0.01 *vs*. LPS group.

Furthermore, the results in Figure 7(a-c) revealed that interference with p65 expression significantly promoted ALP activity and mineralization in LPS-induced hPDLSCs, and Runx2 and Osteopontin expression were significantly upregulated. The above results showed the opposite trend after overexpression of p65.

## Discussion

Studies have shown that the NF-κB signaling pathway regulates osteogenic differentiation of PDLSCs in the inflammatory microenvironment [21,22]. Liu et al. [23] also demonstrated that downregulation of the TRIM52 gene attenuated LPS-induced inflammatory damage in hPDLSCs through the TLR4/NF-κB signaling pathway. Although the important role of NF-κB signaling pathway in periodontitis has been clarified, the specific studies on the involvement of NF-κB signaling pathway in apoptosis and osteogenic differentiation of hPDLSCs after LPS induction need to be further explored. LPS can induce an inflammatory response in hPDLSCs to mimics periodontal disease [24]. Therefore, in this study, with LPS-treated hPDLSCs *in vitro*, the effects and mechanisms of LPS-induced inflammation on hPDLSCs were explored by using NF-κB agonist PMA and inhibitor PDTC, or by direct intervention of NF-κB/p65 expression.

First, hPDLSCs were isolated from tooth root samples, and flow cytometery-based detection of stem cell markers was performed. The results showed that hPDLSCs surface antigens CD146 and STRO-1 were positive, while CD45 expression was negative. CD146 and STRO-1 are two early markers for identification of hPDLSCs, with positive results as judgment criteria [10]. And CD45, a pan-leukocyte marker, is deficient in hPDLSCs, with a negative result as a judgment criterion [25]. Thus, it is suggested that hPDLSCs are successfully isolated from the tooth root samples in our study.

It is well known that NF-κB pathway is considered as a typical pro-inflammatory signaling pathway, mainly based on the activation of NF-κB by pro-inflammatory genes including cytokines, chemokines and adhesion molecules [26]. After identification of hPDLSCs, we then confirmed a significant reduction of proliferation and an increase of apoptosis of hPDLSCs by LPS; inhibition of NF-κB pathway activity or interfering with p65 expression caused suppression of these LPS-induced effects, while activation of this pathway or overexpression of p65 had the opposite effect. Another study have also proved that suppression of NF-κB activity has the inhibitory role in the decrease of proliferation rate and increase of apoptosis rate of hPDLSCs induced by high glucose, and the suppression is also associated with the decreased expression of inflammatory cytokines such as TNF-α and IL-6 [27]. Meng et al. have found that azithromycin inhibits the activation of NF-κB pathway and thereby suppressed apoptosis in hPDLSCs with TNF-α stimulation [28]. Through *in vitro* experiments, we found that inhibition of NF-κB signaling pathway decreased the levels of TNF-ɑ and IL-6 and increased the levels of IL-10 in LPS-induced hPDLSCs.

Osteogenic differentiation of hPDLSCs is diminished in an inflammatory environment [29]. According to the study by Wei et al. [30], Strontium ion reduced expression of pro-inflammatory molecules (TNF-ɑ, IL-1β, and IL-6) in LPS-induced hPDLCs, thereby inhibiting their early osteogenic differentiation. In this study, ALP and alizarin-red staining revealed that inhibition of NF-κB pathway activity or p65 down-regulation was then able to reduce the inhibitory effect of the inflammatory environment on the osteogenic differentiation of hPDLSCs. In addition, inhibition of this pathway could restore LPS-induced down-regulation of Runx2 and osteopontin expression in hPDLSCs. High expression of ALP, Runx2, and osteopontin and high degree of calcium nodules are the markers of osteogenic differentiation [31]. However, some studies have reported that low concentrations of LPS can promote osteogenic differentiation of hPDLSCs [32], and LPS from different bacterial sources has different effects on hPDLSCs [33]. So we speculated that the reason for the discrepancy between the above study and our results may be due to different LPS sources. p65 in the nucleus is an important factor in initiating the inflammatory response and apoptosis program [34]. In this study, we found that LPS resulted in the up-regulation of p-IκBα level in the cytoplasm and the down-regulation of IKKα and IKKβ expression in cell, which in turn promoted the transfer of p65 from the cytoplasm to the nucleus, thereby activating the NF-κB signaling pathway. However, inhibition of NF-κB activation significantly reduced the level of p65 in the nucleus. Yan et al have proved that salvianolic acid C can reduce the nuclear p65, thus inhibiting NF-κB signaling activity [16]. Wang et al. have pointed out that up-regulation of p65 in the nucleus resulted in increased miR-182 expression, which causes damage to hPDLSCs [35]. Collectively, it is suggested that the NF-κB pathway is critical for the inflammation-induced destruction in hPDLSCs. However, the present study also has limitations. Because of the complex inflammatory environment of periodontitis, how NF-κB pathway affects hPDLSCs differentiation ability has not been studied in detail, and the mechanism of its effect on periodontitis *in vivo* needs to be investigated in depth.

## Conclusion

In summary, activation of NF-κB pathway activity inhibits the proliferation and differentiation ability of LPS-treated hPDLSCs, and leads to increased release of TNF-α and IL-6 and reduced production of IL-10. Therefore, avoiding the over-activation response of this pathway can improve the efficacy of clinical application or implantation of bioengineered hPDLSCs.

## Highlights

1. LPS induces apoptosis of hPDLSCs and inhibits osteogenic differentiation.
2. LPS activates NF-κB signaling pathway in hPDLSCs.
3. NF-κB pathway inhibitor PDTC inhibits LPS-induced apoptosis of hPDLSCs and promoted osteogenic differentiation.
4. PDTC inhibits LPS-induced inflammatory response.

## References

[1] Bansal M, Mittal N, Singh TB. Assessment of the prevalence of periodontal diseases and treatment needs: a hospital-based study. J Indian Soc Periodontol. 2015;19(2):211–215. 26015675 10.4103/0972-124X.145810 PMC4439634

[2] Delange N, Lindsay S, Lemus H, et al. Periodontal disease and its connection to systemic biomarkers of cardiovascular disease in young American Indian/Alaskan natives. J Periodontol. 2018;89:219–227. 29520828 10.1002/JPER.17-0319 PMC6242269

[3] Jiang H, Zhang Y, Xiong X, et al. Salivary and serum inflammatory mediators among pre-conception women with periodontal disease. BMC Oral Health. 2016;16(1):131. 27978823 10.1186/s12903-016-0306-9 PMC5159988

[4] Michaud DS, Fu Z, Shi J, et al. Periodontal disease, tooth loss, and cancer risk. Epidemiol Rev. 2017;39(1):49–58. 28449041 10.1093/epirev/mxx006 PMC5868279

[5] Nazir MA. Prevalence of periodontal disease, its association with systemic diseases and prevention. Int J Health Sci (Qassim). 2017;11(2):72–80. 28539867 PMC5426403

[6] Fiedler T, Salamon A, Adam S, et al. Impact of bacteria and bacterial components on osteogenic and adipogenic differentiation of adipose-derived mesenchymal stem cells. Exp Cell Res. 2013;319(18):2883–2892. 23988607 10.1016/j.yexcr.2013.08.020

[7] Herzmann N, Salamon A, Fiedler T, et al. Lipopolysaccharide induces proliferation and osteogenic differentiation of adipose-derived mesenchymal stromal cells in vitro via TLR4 activation. Exp Cell Res. 2017;350(1):115–122. 27865937 10.1016/j.yexcr.2016.11.012

[8] Cochran DL. Inflammation and bone loss in periodontal disease. J Periodontol. 2008;79(8s):1569–1576. 18673012 10.1902/jop.2008.080233

[9] Gu K, Fu X, Tian H, et al. TAZ promotes the proliferation and osteogenic differentiation of human periodontal ligament stem cells via the p-SMAD3. J Cell Biochem. 2020;121(2):1101–1113. 31478222 10.1002/jcb.29346

[10] Seo BM, Miura M, Gronthos S, et al. Investigation of multipotent postnatal stem cells from human periodontal ligament. Lancet. 2004;364(9429):149–155. 15246727 10.1016/S0140-6736(04)16627-0

[11] Xu J, Wang W, Kapila Y, et al. Multiple differentiation capacity of STRO-1+/CD146+PDL mesenchymal progenitor cells. Stem Cells Dev. 2009;18(3):487–496. 18593336 10.1089/scd.2008.0113 PMC2702120

[12] Shi S, Bartold PM, Miura M, et al. The efficacy of mesenchymal stem cells to regenerate and repair dental structures. Orthod Craniofac Res. 2005;8(3):191–199. 16022721 10.1111/j.1601-6343.2005.00331.x

[13] Mrozik KM, Wada N, Marino V, et al. Regeneration of periodontal tissues using allogeneic periodontal ligament stem cells in an ovine model. Regen Med. 2013;8(6):711–723. 24147527 10.2217/rme.13.66

[14] Baksh D, Song L, Tuan RS. Adult mesenchymal stem cells: characterization, differentiation, and application in cell and gene therapy. J Cell Mol Med. 2004;8(3):301–316. 15491506 10.1111/j.1582-4934.2004.tb00320.x PMC6740223

[15] Liu N, Shi S, Deng M, et al. High levels of β-catenin signaling reduce osteogenic differentiation of stem cells in inflammatory microenvironments through inhibition of the noncanonical Wnt pathway. J Bone Miner Res. 2011;26(9):2082–2095. 21638320 10.1002/jbmr.440

[16] Duan Y, An W, Wu H, et al. Salvianolic acid C attenuates LPS-induced inflammation and apoptosis in human periodontal ligament stem cells via toll-like receptors 4 (TLR4)/nuclear factor kappa B (NF-κB) pathway. Med Sci Monit. 2019;25:9499–9508. 31831723 10.12659/MSM.918940 PMC6929551

[17] Islam SU, Lee JH, Shehzad A, et al. Decursinol angelate inhibits LPS-induced macrophage polarization through modulation of the NFκB and MAPK signaling pathways. Molecules. 2018;23:1880. 10.3390/molecules23081880 PMC6222640 30060484

[18] Zhu HP, Huang HY, Wu DM, et al. Regulatory mechanism of NOV/CCN3 in the inflammation and apoptosis of lung epithelial alveolar cells upon lipopolysaccharide stimulation. Mol Med Rep. 2020;21(4):1872–1880. 31545412 10.3892/mmr.2019.10655 PMC7057825

[19] Ali M, Yang F, Jansen JA, et al. Lipoxin suppresses inflammation via the TLR4/MyD88/NF-κB pathway in periodontal ligament cells. Oral Dis. 2020;26(2):429–438. 31814225 10.1111/odi.13250 PMC7074052

[20] Kanazawa I, Yamaguchi T, Yano S, et al. Adiponectin and AMP kinase activator stimulate proliferation, differentiation, and mineralization of osteoblastic MC3T3-E1 cells. BMC Cell Biol. 2007;8(1):51. 18047638 10.1186/1471-2121-8-51 PMC2214728

[21] Chen X, Hu C, Wang G, et al. Nuclear factor-κB modulates osteogenesis of periodontal ligament stem cells through competition with β-catenin signaling in inflammatory microenvironments. Cell Death Dis. 2013;4(2):e510. 23449446 10.1038/cddis.2013.14 PMC3734811

[22] Yu B, Li Q, Zhou M. LPS‑induced upregulation of the TLR4 signaling pathway inhibits osteogenic differentiation of human periodontal ligament stem cells under inflammatory conditions. Int J Mol Med. 2019;43(6):2341–2351. 31017254 10.3892/ijmm.2019.4165 PMC6488177

[23] Liu P, Cui L, Shen L. Knockdown of TRIM52 alleviates LPS-induced inflammatory injury in human periodontal ligament cells through the TLR4/NF-κB pathway. Biosci Rep. 2020;40(8):BSR20201223. 32735017 10.1042/BSR20201223 PMC7418211

[24] Chen W, Su J, Cai S, et al. Cullin3 aggravates the inflammatory response of periodontal ligament stem cells via regulation of SHH signaling and Nrf2. Bioengineered. 2021;12(1):3089–3100. 34193016 10.1080/21655979.2021.1943603 PMC8806625

[25] Vasandan AB, Shankar SR, Prasad P, et al. Functional differences in mesenchymal stromal cells from human dental pulp and periodontal ligament. J Cell Mol Med. 2014;18(2):344–354. 24393246 10.1111/jcmm.12192 PMC3930420

[26] Lawrence T. The nuclear factor NF-kappaB pathway in inflammation. Cold Spring Harb Perspect Biol. 2009;1(6):a001651. 20457564 10.1101/cshperspect.a001651 PMC2882124

[27] Kato H, Taguchi Y, Tominaga K, et al. High glucose concentrations suppress the proliferation of human periodontal ligament stem cells and their differentiation into osteoblasts. J Periodontol. 2016;87(4):e44–51. 26537370 10.1902/jop.2015.150474

[28] Meng T, Zhou Y, Li J, et al. Azithromycin promotes the osteogenic differentiation of human periodontal ligament stem cells after stimulation with TNF-α. Stem Cells Int. 2018;2018:7961962. 30515223 10.1155/2018/7961962 PMC6234456

[29] Diomede F, Thangavelu SR, Merciaro I, et al. Porphyromonas gingivalis lipopolysaccharide stimulation in human periodontal ligament stem cells: role of epigenetic modifications to the inflammation. Eur J Histochem. 2017;61(3):2826. 29046054 10.4081/ejh.2017.2826 PMC5575416

[30] Wei L, Jiang Y, Zhou W, et al. Strontium ion attenuates lipopolysaccharide-stimulated proinflammatory cytokine expression and lipopolysaccharide-inhibited early osteogenic differentiation of human periodontal ligament cells. J Periodontal Res. 2018;53(6):999–1008. 30221352 10.1111/jre.12599

[31] Jia Q, Jiang W, Ni L. Down-regulated non-coding RNA (lncRNA-ANCR) promotes osteogenic differentiation of periodontal ligament stem cells. Arch Oral Biol. 2015;60(2):234–241. 25463901 10.1016/j.archoralbio.2014.10.007

[32] Tang J, Wu T, Xiong J, et al. Porphyromonas gingivalis lipopolysaccharides regulate functions of bone marrow mesenchymal stem cells. Cell Prolif. 2015;48(2):239–248. 25676907 10.1111/cpr.12173 PMC6496502

[33] Huang Y, Jiang H, Gong Q, et al. Lipopolysaccharide stimulation improves the odontoblastic differentiation of human dental pulp cells. Mol Med Rep. 2015;11(5):3547–3552. 25528991 10.3892/mmr.2014.3120

[34] Čebatariūnienė A, Kriaučiūnaitė K, Prunskaitė J, et al. Extracellular vesicles suppress basal and lipopolysaccharide-induced NFκB activity in human periodontal ligament stem cells. Stem Cells Dev. 2019;28(15):1037–1049. 31017040 10.1089/scd.2019.0021

[35] Wang L, Wu F, Song Y, et al. Long noncoding RNA related to periodontitis interacts with miR-182 to upregulate osteogenic differentiation in periodontal mesenchymal stem cells of periodontitis patients. Cell Death Dis. 2016;7(8):e2327. 27512949 10.1038/cddis.2016.125 PMC5108307

